# Dosimetric predictors of nephrotoxicity in patients receiving extended-field radiation therapy for gynecologic cancer

**DOI:** 10.1186/s13014-021-01755-z

**Published:** 2021-02-04

**Authors:** Hiroaki Kunogi, Nanae Yamaguchi, Yasuhisa Terao, Keisuke Sasai

**Affiliations:** 1 Department of Radiation Oncology, Juntendo University, 2-1-1, Hongo, Bunkyo-ku, Tokyo, 113-8421 Japan; 2 Department of Gynecology, Juntendo University, Tokyo, Japan

**Keywords:** Extended-field radiation therapy, Gynecological oncology, Nephrotoxicity

## Abstract

**Purpose:**

We sought dosimetric predictors of a decreasing estimated glomerular filtration rate (eGFR) in gynecological oncology patients receiving extended-field radiation therapy (EFRT).

**Materials and methods:**

Between July 2012 and April 2020, 98 consecutive cervical or endometrial cancer patients underwent EFRT or whole-pelvis radiation therapy (WPRT) with concurrent cisplatin chemotherapy in our institution. To explore the effect of concurrent cisplatin chemotherapy on renal function, the renal function of the WPRT patients was examined. Of the 98 patients, 34 cervical or endometrial cancer patients underwent EFRT including extended-field intensity-modulated radiation therapy (EF-IMRT) and 64 cervical cancer patients underwent WPRT with cisplatin. Of the 34 EFRT patients, 32 underwent concurrent cisplatin chemotherapy. Excluding patients exhibiting recurrences within 6 months, 31 EFRT patients were analyzed in terms of the dose-volume kidney histograms (the percentages of kidney volumes receiving 12, 16, 20, and 24 Gy) and the post- to pre-treatment eGFR ratios. We calculated Pearson correlation coefficients between the renal dose volume and the percentage eGFR reductions of the 31 EFRT patients, and those treated via EF-IMRT. Renal dose constraint significance was evaluated using the Mann–Whitney U test.

**Results:**

The eGFR value after WPRT with cisplatin remained largely unchanged for 12 months, unlike that after EFRT. In EFRT patients, a strong correlation was evident between the KV_20Gy_ dose and the post- to pre-treatment eGFR ratio (correlation coefficients − 0.80 for all patients and − 0.74 for EF-IMRT patients). In EF-IMRT patients, the kidney volume receiving 20 Gy tended to correlate negatively with the eGFR reduction. The Mann–Whitney U test showed that patients with KV_20Gy_ values < 10% retained significantly better renal function than did patients with KV_20Gy_ values > 10% (*P* = 0.002).

**Conclusions:**

Imposition of a severe kidney dose constraint during EF-IMRT may reduce nephrotic toxicity. Future prospective investigations of kidney-sparing EF-IMRT are required.

## Introduction

Extended-field radiation therapy (EFRT) targeting all of the pelvic and para-aortic lymph nodes effectively treats patients with advanced cervical or endometrial cancer [1–7], but is associated with a risk of renal dysfunction [8]. The kidney constraints imposed were a maximum of 45 Gy, a maximum V_16Gy_ of 35%, and a decrease in the mean initial creatinine clearance (CrCl) of 17.6% [8]. Renal dose reduction is required during EFRT planning.

Few reports have explored the relationship between dose volumes to the kidney during EFRT and the extent of associated nephrotoxicity. Elucidation of this relationship would aid definition of an optimal renal dose constraint. It is essential to avoid nephrotic toxicity, particularly in patients with long life expectancies. We used EFRT (including kidney-sparing non-coplanar EF-IMRT [9]) to treat patients with cervical or endometrial cancer. We retrospectively investigated the relationship between the kidney dose-volume histograms (DVHs) and changes in renal function. To assess the effect of concurrent cisplatin chemotherapy on renal function, the renal function of patients who underwent whole-pelvis radiation therapy (WPRT, without kidney irradiation) was examined retrospectively.

## Materials and methods

### Patient population

The clinical data of 98 consecutive patients treated via definitive EFRT (pelvic plus para-aortic radiotherapy, 50.4 Gy in 28 fractions with a sequential additional boost to treat involved nodes) or via WPRT with concurrent cisplatin chemotherapy (50.4 Gy in 28 fractions, with or without a sequential additional nodal boost) between July 2012 and April 2020 in our institution were retrospectively reviewed. In our institution, EFRT is used when nodal disease has extended to the para-aortic or common iliac nodes on positron emission tomography with ^18^F-labeled fluoro-2-deoxyglucose/computed tomography (^18^F-FDG-PET/CT). Of these 98 patients, 34 cervical or endometrial cancer patients underwent EFRT, and 64 cervical cancer patients underwent WPRT with concurrent cisplatin chemotherapy. The 34 EFRT patients (primary cervical cancer n = 32, postoperative recurrent cervical cancer n = 1, and primary endometrial cancer n = 1) and 64 WPRT patients completed their treatment courses. Prior to treatment, written informed consent was obtained from all patients, who agreed to our use of their clinical data. This retrospective study was approved by the ethics committee of our institution (Approval No. 17-291) which waived the need for re-informed consent. Of the 34 EFRT patients, 32 (94%) underwent concurrent cisplatin chemotherapy. When evaluating the renal toxicity of EFRT, patients developing recurrences within 6 months were excluded. Ultimately, 31 EFRT patients were analyzed in terms of the kidney DVHs and changes in renal function. Of the 64 WPRT patients, the renal function of the 48 patients who were followed with no recurrence for more than 12 months was examined.

### Radiotherapy

**Fig. 1:**
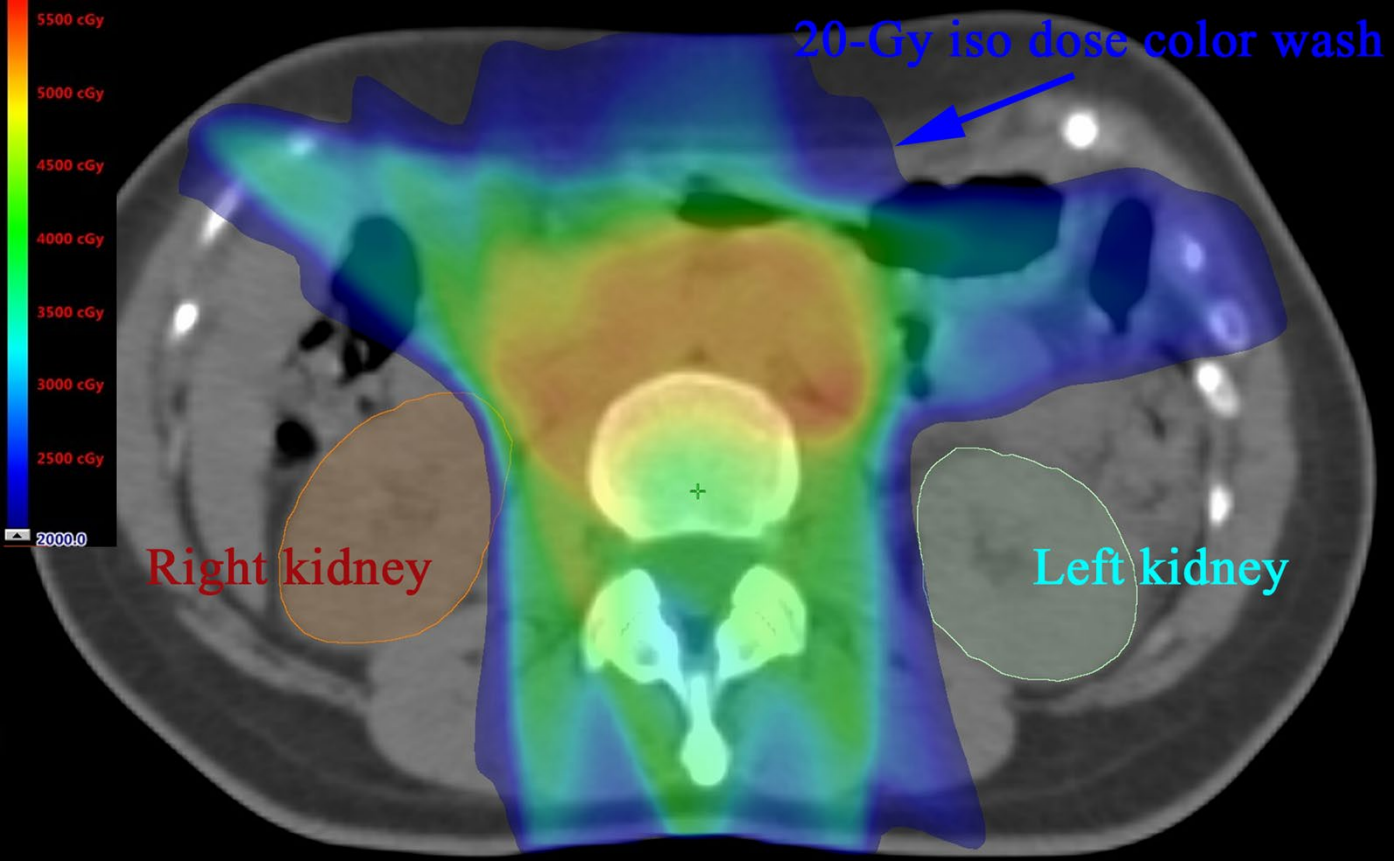
Example axial images showing the isodose distributions for the EF-IMRT plans of a representative patient. The volume percentage was 1.9% for both kidneys that received 20 Gy and there was no renal dysfunction. The parts of the right (brown line) and left (cyan line) kidneys excluded from the 20-Gy isodose color wash (blue) are shown

During external beam planning, the median computed tomography (CT) spacing was 3 mm (3–5 mm) under natural respiration. The clinical target volume (CTV) included a primary CTV and nodal CTV, including the pelvic and para-aortic lymph nodes. The pelvic lymph nodes were delineated by reference to the Japan Clinical Oncology Group Gynecologic Cancer Study Group (JCOG-GCSG) consensus guidelines for such delineation [10]. The para-aortic lymph nodes included those in the region between the psoas muscles, superiorly above the level of the renal artery, and anteriorly in the area encompassed by the aorta and inferior vena cava with a 7 mm margin. In patients with positive nodes just below the celiac vessel, the para-aortic region was contoured to include the region superior to that vessel. In all patients, the CTV was isotropically expanded by 5–7 mm (median 7 mm) to create the planning target volume (PTV). Using the normal tissue contouring guidelines [11, 12], organs at risk (OARs) including the small bowel (contoured as a peritoneal space), both kidneys, and the spinal cord were delineated. No margin was added to the contoured OARs. All patients were irradiated with 10-MV photons. The type of EBRT used changed over the course of the study. Box-field EFRT plans were generated for gantry angles of 0°, 90°, 180°, and 270° using the field-in-field technique without OAR dose constraints, including kidney dose constraints. Most EF-IMRT plans were generated using seven fixed fields, and the gantry angles of most plans were 50°, 85°, 160°, 180°, 200°, 275°, and 310°. The EF-IMRT plans were optimized using the “Normal Tissue Objective” function of the Eclipse Planning System (Varian Medical Systems, Palo Alto, CA, USA) to spare the OARs (small bowel, bladder, rectum, and spinal cord). The kidney priority was lower than those of the PTV, bowel, and spinal cord during EF-IMRT planning. A plan was accepted if 95% of the PTV volume received 98% of the prescribed dose, with the maximum dose being < 110%, and 98% of the PTV received 95% of the prescribed dose, while keeping the irradiated volumes delivered to the small bowel, which received 40 Gy, and the bladder/rectum as low as possible. We aimed to meet the spinal cord (≤ 0.1 cc at 45 Gy) and kidney (≤ 35% at 16 Gy) constraints of Gerszten et al. and Varlotto et al. [1, 8]. Beginning in April 2016, we aimed to reduce kidney exposure by reconfiguring the low-dose constraints (10–20 Gy) of the kidneys, using non-coplanar beams when necessary. In six patients, EF-IMRT plans featuring kidney-sparing non-coplanar beams were created to reduce the irradiated kidney volume; we have reported the procedure previously [9]. Briefly, non-coplanar EF-IMRT plans were generated using seven non-coplanar beams, i.e., a combination of three coplanar anterior beams, two lateral inferior oblique beams, and two posterior inferior oblique beams. A coplanar EF-IMRT plan was initially created with PTV coverage and sparing of OARs. Next, oblique beams were designed to reduce irradiation of the kidney volume at the same gantry angles as used in the coplanar plan, such as gantry/couch angles of 85°/340°, 275°/20°, 160°/340°, or 20°/20°. The low-dose (10–20 Gy) irradiated kidney volumes were kept as low as possible by oblique beams, without compromising the PTV coverage and sparing of other OARs. Figure 1 shows axial images of the EF-IMRT plan at the kidney level for a representative patient. When creating the sequential boost plans (either 5.4 Gy in three fractions or 9 Gy in five fractions) for involved nodes, including para-aortic nodes, the incident beams did not traverse the kidney. If the small bowel was not included in the treatment volume, 9 Gy was used in five fractions. Daily positioning verification was performed using two-dimensional matching or cone-beam CT matching. Two-dimensional matching was done using electronic portal images (two orthogonal views) and digitally reconstructed (from the planning CT) radiographs of bony structures. Cone-beam CT matching was performed using bony structures. Two-dimensional matching or cone-beam CT matching changed over the course of the study. Of the 34 EFRT patients, 33 received high-dose-rate (HDR) brachytherapy to treat each primary gynecological site.

### Patient follow-up

After treatment, patients were followed-up (without adjuvant therapy) every 1–2 months for 3 years and every 3 or 4 months thereafter. Serum creatinine measurements were performed at each visit to evaluate renal function. Toxicities were evaluated using the Common Terminology Criteria for Adverse Events ver. 4.0 (CTCAE-4) [13].

### Retrospective evaluation of renal dosimetry

To verify retrospectively the validity of the kidney DVH parameters, we evaluated both kidneys together, although the right and left kidneys were separately considered during treatment planning. The metrics calculated were the KV_12Gy_, KV_16Gy_, KV_20Gy_, and KV_24Gy_ (i.e., the percent volumes of both kidneys receiving 12 Gy, 16 Gy, 20 Gy, and 24 Gy, respectively). The HDR brachytherapy doses were excluded from renal dosimetric analyses, because the kidneys lay distant from the primary sites.

### The timing of renal function reductions after EFRT

We reviewed the renal function data of patients followed-up with no recurrence for more than 12 months to time all falls in renal function after EFRT. In 23 of the 34 patients, the percentages of the post-treatment estimated glomerular filtration rates (eGFR) compared to the pre-treatment eGFRs (taken to be 100%) were calculated. Each eGFR was estimated using the following equation for Japanese subjects [14]:

$${\text{eGFR}}\,\left( {{\text{mL/min/}}1.73\,{\text{m}}^{2} } \right) = 194 \times {\text{serum creatinine}}\,\left( {\text{mg/dL}} \right)^{{{-}1.094}} \times {\text{age}}^{{{-}0.287}} \times 0.739.$$

### Confounding factors in the analysis of renal function reductions

To examine factors confounding the renal function reduction results, we compared EFRT patients with and without a medical history likely to affect renal function. To assess the effect of concurrent cisplatin chemotherapy on renal function, we also examined the post- to pre-treatment eGFR ratios of 48 cervical cancer patients, who were followed without recurrence for more than 12 months after WPRT with cisplatin, and compared the renal function reductions at 6 months between the EFRT and WPRT groups.

### Statistics

**Fig. 2:**
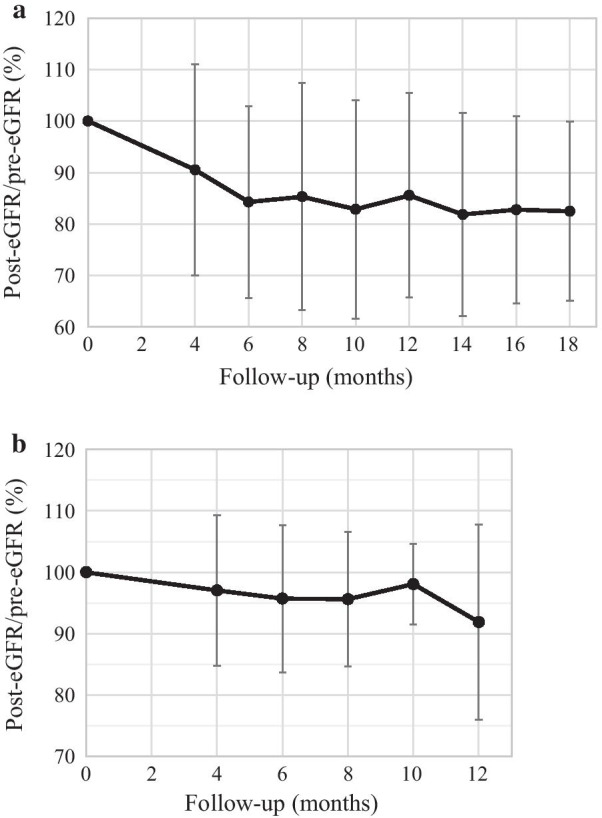
The ratios of post- to pre-treatment eGFR in the EFRT (**a**) and WPRT (**b**) groups. Mean ratios are shown by the time elapsed from the first day of treatment. In the EFRT group, the eGFR tended to decline until 6 months and then remained largely unchanged. In the WPRT group, the eGFR remained largely unchanged. Each error bar indicates one standard deviation of the mean. *eGFR* estimated glomerular filtration rate

We calculated Pearson correlation coefficients between each of the KV_12Gy_/KV_16Gy_/KV_20Gy_/KV_24Gy_ and the eGFR reductions to determine the renal dose-volume predicting reduced renal function. Based on the timing of such reductions (Fig. 2a), the percentage eGFRs at 6 months were calculated for all 31 patients. We rounded off the median ratios of post- to pre-treatment eGFRs. The significance of each obtained kidney dose constraint was evaluated using the Mann–Whitney U test. We also compared EFRT patients with and without a medical history affecting renal function, and compared the reductions in renal function at 6 months in the two groups, again using the Mann–Whitney U test. All statistical analyses were performed using SPSS ver. 18 (SPSS Inc., Chicago, IL, USA). A *P-*value less than 0.05 was considered to indicate statistical significance.

## Results

### Patient characteristics

Table 1 lists the patient characteristics. The median follow-up time after the first day of treatment was 24 months (range 8–100 months) for 31 EFRT patients, thus excluding the three who developed recurrences within 6 months. The median follow-up time for 9 patients with a medical history potentially affecting renal function was 20 months (range 8–100 months).

**Table 1:** Patient and treatment characteristics in the EFRT (A) and WPRT (B) groups

(A)	
EFRT patient characteristics	
*Age (years)*	
Mean (SD)	54.8 (13.8)
Median (range)	52 (35–85)
Primary cervical cancer	32 (94%)
Post-operative recurrent cervical cancer	1 (3%)
Primary endometrial cancer	1 (3%)
*Medical history affecting renal function*	
None	25 (74%)
Hypertension	5 (15%)
Hypercholesterolemia	1 (3%)
Diabetes and hypercholesterolemia	1 (3%)
Diabetes, hypertension, and hypercholesterolemia	1 (3%)
Nephrotic syndrome	1 (3%)
*Pre-treatment baseline creatinine clearance*	
Mean (SD)	86.6 mL/min (20.5)
Median (range)	84 mL/min (57.8–149.8)
*Histology*	
Squamous cell carcinoma	28 (82%)
Adenocarcinoma	6 (18%)
*Para-aortic node positive on *^*18*^*F-FDG-PET/CT*	
Yes	32 (94%)
No	2 (6%)
*External beam radiotherapy*	
Box field EFRT	13 (38%)
Coplanar EF-IMRT	15 (44%)
Non-coplanar EF-IMRT	6 (18%)
*EFRT dose (Gy)*	
Median (range)	55.8 (55.8–59.4)

(B)	
WPRT patient characteristics	
*Age (years)*	
Mean (SD)	57.1 (12.9)
Median (range)	56 (31–85)
Primary cervical cancer	54 (84%)
Post-operative cervical cancer	10 (16%)
*Medical history affecting renal function*	
None	49 (77%)
Hypertension	6 (9%)
Hypercholesterolemia	5 (8%)
Diabetes and hypertension	2 (3%)
Diabetes	1 (2%)
Hypertension and hypercholesterolemia	1 (2%)
*Pre-treatment baseline creatinine clearance*	
Mean (SD)	83.4 mL/min (19.1)
Median (range)	80.8 mL/min (47.6–152.1)

*EF-IMRT* extended-field intensity-modulated radiation therapy, *EFRT* extended-field radiation therapy, ^*18*^*F-FDG-PET/CT* positron emission tomography with ^18^F-labeled fluoro-2-deoxyglucose/computed tomography, *SD* standard deviation, *WPRT* whole-pelvis radiation therapy

### Timing of the fall in renal function after EFRT

The mean ratios of the post- to pre-treatment eGFRs are shown by elapsed time (from the first day of EFRT) in Fig. 2a. The eGFR tended to decline up to 6 months and then remained largely unchanged. Therefore, the 6-month eGFRs were used for analysis of nephrotoxic dosimetry.

### Confounding factors in the analysis of renal function reductions

The Mann–Whitney U test showed that the change in eGFR did not differ significantly between the patients with and without a medical history affecting renal function (mean change in eGFR: 91% vs. 81%, *P* = 0.19). The renal function data for 48 cervical cancer patients after WPRT with cisplatin is shown in Fig. 2b. The mean eGFR remained largely unchanged for 12 months (mean eGFR reduction = 4% at 6 months). The renal functions of the 31 EFRT patients differed significantly from those of the 48 WPRT patients 6 months after the first day of treatment (*P* = 0.014).

### Correlations between irradiated renal volumes and eGFR reduction percentages

**Fig. 3:**
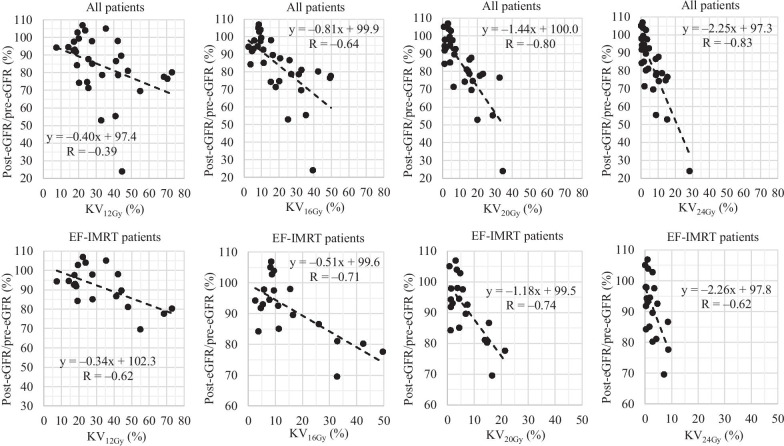
The associations between the KV_12Gy_/KV_16Gy_/KV_20Gy_/KV_24Gy_ and the percentage eGFR reductions at 6 months. *eGFR* estimated glomerular filtration rate. EF-IMRT, extended-field intensity-modulated radiation therapy; EFRT, extended-field radiation therapy. KV_12Gy_, KV_16Gy_, KV_20Gy_, and KV_24Gy_ = percentage volumes of kidney receiving 12 Gy, 16 Gy, 20 Gy, and 24 Gy, respectively

The associations between the KV_12Gy_/KV_16Gy_/KV_20Gy_/KV_24Gy_ and eGFR reductions (the pre-treatment percentages were taken to be 100%) are plotted in Fig. 3. An (approximate) inverse linear relationship was evident. Table 2 lists the correlation coefficients and the slopes of the (approximately) straight lines. For all 31 patients, the KV_20Gy_ and KV_24Gy_ data yielded higher correlation coefficients, and the KV_20Gy_ line slope was closer to -1 than was the KV_24Gy_ slope. For EF-IMRT patients, the KV_16Gy_ and KV_20Gy_ data yielded higher correlation coefficients, and the KV_20Gy_ line slope was closer to -1 than was the KV_16Gy_ slope. Thus, the KV_20Gy_ data may predict the eGFR decrease. In all KV_20Gy_ graphs (Fig. 3), the horizontal dividing percentage (the rounded-off median eGFR percentage) was 85%. The dividing rounded- off value of KV_20Gy_ associated with the rounded-off median eGFR percentage (85%) was 10%. The Mann–Whitney U test indicated that patients with KV_20Gy_ values < 10% retained significantly better renal function than did patients with KV_20Gy_ values > 10% (*P* = 0.002).

**Table 2:** Correlation coefficients and the slopes of the (approximately) straight lines

	R^a^	Slopes
*All patients*		
KV_12Gy_	− 0.39	− 0.40
KV_16Gy_	− 0.64	− 0.81
KV_20Gy_	− 0.80	− 1.44
KV_24Gy_	− 0.83	− 2.25
*EF-IMRT patients*		
KV_12Gy_	− 0.62	− 0.34
KV_16Gy_	− 0.71	− 0.51
KV_20Gy_	− 0.74	− 1.18
KV_24Gy_	− 0.62	− 2.26

^a^Correlation coefficients

*EF-IMRT* extended-field intensity-modulated radiation therapy, *KV*_*12Gy*_*, KV*_*16Gy*_*, KV*_*20Gy*_*, and KV*_*24Gy*_ percentage volumes of kidney receiving 12 Gy, 16 Gy, 20 Gy, and 24 Gy, respectively

## Discussion

We demonstrate here the relationship between the kidney dose-volume data and the extent of associated nephrotoxicity. This is the first study to explore the possibility of establishing an optimal renal dose constraint for EF-IMRT, which was determined to be KV_20Gy_ < 10%. This reduces the eGFR percentage, because the slope of the graph is close to -1. This may be critically important. In a clinical setting, the KV_20Gy_ of the EF-IMRT DVH may represent the reduction in the eGFR about 6 months after.

The eGFR in most of our patients was within the normal range after EFRT. A strict kidney dose constraint (KV_20Gy_ < 10%) is not intended to prevent renal failure (i.e., renal toxicity ≥ grade 3), but rather to lower the risk of nephrotoxicity in association with EFRT. Kidney-sparing may be particularly important in patients with a long life expectancy.

OAR prioritization during EF-IMRT planning remains variable. It is sometimes difficult to satisfy every normal tissue dose constraint without compromising the PTV coverage, because the extent of kidney irradiation during EFRT depends on the anatomical locations of the kidneys with respect to the PTV and the other OARs. During our EF-IMRT planning, the kidney priority was lower than that of the PTV, bowel, and spinal cord. In a clinical setting, it may be difficult to satisfy a demanding kidney dose constraint without compromising PTV coverage and the other OARs. The priority depends on the judgment of the radiation oncologist.

Hydronephrosis caused by radiation-induced ureter stenosis [15] may trigger renal dysfunction; we did not encounter this problem.

To spare functional kidneys, functional imaging may aid future EF-IMRT planning. Our dosimetric predictors of nephrotoxicity were archived; we assumed that renal function before treatment would reflect the delineated kidney volume derived using the normal tissue contouring guidelines [12]. The dosimetric predictors of nephrotoxicity were also archived; we assumed that the left and right kidneys would exhibit the same renal function/volume parameters. If the renal function of the left kidney were to differ from that of the right kidney (perhaps because of renal vascular sclerosis), functional kidney imaging may be required. Future, advanced imaging techniques may allow incorporation of functional kidney information into EF-IMRT planning.

The internal kidney margins during EF-IMRT planning require attention. Kidneys can move during natural respiration. Indeed, our EFRT planning CT was performed under natural respiration; no margins were added to the contoured OARs. Measurements of kidney motion either under natural respiration or on breath-holding are available [16, 17]. The use of OAR volumes featuring appropriate margins is desirable.

No optimal eGFR reduction percentage is yet available; this would aid definition of the nephrotoxic effects of radiotherapy. Our cutoff percentage was 15%, based on the rounded-off median eGFR percentage after treatment. As shown in Fig. 2a, the mean eGFR reduction percentage was 16% at 6 months after treatment, and the mean initial CrCl decreased by 17.6% after para-aortic radiotherapy [8]. However, in the clinic, slight dehydration may develop in patients lacking symptoms, who thus experience 5–10% drops in the eGFR. A reduction percentage of 15% may be reasonable in practice.

A limitation of this study is that it was retrospective. Commencing in April 2016, we sought to deliver no more than 10–20 Gy to the kidneys. EF-IMRT plans that included kidney-sparing non-coplanar subplans [9] were carefully generated without compromising PTV coverage; we also spared other OARs. Future, prospective, kidney-sparing prospective EF-IMRT studies are needed.

Another limitation of this study is that the renal dosimetric analyses were performed according to the serum creatinine level at 6 months. Based on the results shown in Fig. 2a, the 6-month eGFR data used in the analysis of nephrotoxic dosimetry seemed to be appropriate, although the maximum radiation-induced eGFR reduction may not be at exactly 6 months.

Other factors may also be associated with change in eGFR, including the medical history and cisplatin use. In our study, there was no significant difference in the change in eGFR according to medical history. In the 48 patients receiving WPRT in our institution, the mean eGFR remained largely unchanged for 12 months (mean eGFR reduction = 4% at 6 months), as shown in Fig. 2b. However, EFRT patients generally receive concurrent cisplatin chemotherapy, and cisplatin may affect the reduction in eGFR. Our results may apply mainly to patients receiving EFRT with cisplatin.

An effect of doses below 20 Gy on the kidneys has been documented. Nineteen percent of patients with Wilms’ tumors who received a low dose (less than 12 Gy) exhibited impaired CrCl [18]. Over 40% of nine children who received 8–12 Gy of total body irradiation developed reduced eGFRs by 5 years of follow-up [19]. This study retrospectively analyzed only a small number of patients, so it is difficult to draw definitive conclusions. Further studies are required to determine if the 20-Gy kidney-sparing dose constraint is excessive. If it is very easy to reduce the KV_12Gy_ and/or KV_16Gy_ values, this may be useful. Dosimetric analysis to reduce the risk of EFRT-induced renal hypertension is also required. The risk of renal hypertension after radiotherapy is well-known [20]; one of nine children who received 8–12 Gy of total body irradiation developed hypertension 13 years later [19]. In our study, five patients were hypertensive prior to EFRT but only one developed hypertension 87 months later. The KV12Gy, KV16Gy, KV20Gy, and KV24Gy were 25.7%, 20.7%, 16.6%, and 10.3%, respectively. It is unclear whether this hypertension was attributable to radiation nephropathy. It may be premature to evaluate radiation-induced renal hypertension status after a median follow-up time of only 24 months. We found it difficult to define optimal dose constraints avoiding EFRT-induced renal hypertension.

## Conclusions

The use of a KV_20Gy_ < 10% during EF-IMRT planning may reduce nephrotic toxicity. Future investigation of kidney-sparing EF-IMRT is needed, as is a prospective study evaluating efficacy.

## Data Availability

Not applicable.

## Abbreviations

CrCl: Creatinine clearance
CT: Computed tomography
CTCAE-4: Common Terminology Criteria for Adverse Events version 4.0
CTV: Clinical target volume
DVH: Dose-volume histogram
EF-IMRT: Extended-field intensity-modulated radiation therapy
EFRT: Extended-field radiation therapy
eGFR: Estimated glomerular filtration rate
HDR: High-dose-rate
JCOG-GCSG: Japan Clinical Oncology Group Gynecologic Cancer Study Group
KV_12Gy_, KV_16Gy_, KV_20Gy_, and KV_24Gy_: Percentage volumes of both kidneys receiving 12 Gy, 16 Gy, 20 Gy, and 24 Gy, respectively
OARs: Organs at risk
^18^F-FDG-PET/CT: Positron emission tomography with ^18^F-labeled fluoro-2-deoxyglucose/computed tomography
PTV: Planning target volume
WPRT: Whole-pelvis radiation therapy
